# Age-related GABA- and glutamatergic differences in SMA during bimanual coordination

**DOI:** 10.1162/IMAG.a.1036

**Published:** 2025-11-26

**Authors:** Joana Frieske, Sara Magalhães Ferreira, Shanti Van Malderen, Melina Hehl, Richard A.E. Edden, Stephan P. Swinnen, Raf L.J. Meesen, Koen Cuypers

**Affiliations:** Neuroplasticity and Movement Control Research Group, Rehabilitation Research Institute (REVAL), Hasselt University, Diepenbeek, Belgium; Movement Control & Neuroplasticity Research Group, Department of Movement Sciences, Group Biomedical Sciences, KU Leuven, Heverlee, Belgium; Leuven Brain Institute (LBI), KU Leuven, Belgium; Department of Imaging and Pathology, Group Biomedical Sciences, KU Leuven, Leuven, Belgium; Russell H. Morgan Department of Radiology and Radiological Science, The Johns Hopkins University School of Medicine, Baltimore, MD, United States; F. M. Kirby Research Center for Functional Brain Imaging, Kennedy Krieger Institute, Baltimore, MD, United States

**Keywords:** MRS, SMA, motor control, aging, GABA, Glx

## Abstract

The Supplementary Motor Area (SMA) is key in bimanual coordination and higher order motor control. However, little is known about how excitatory and inhibitory neurometabolites in this region relate to motor performance, particularly across the adult lifespan. Using magnetic resonance spectroscopy (MRS), we investigated age-related differences in gamma-aminobutyric acid (GABA+) and glutamate + glutamine (Glx) levels in the SMA and their relationship with bimanual motor performance. Twenty-seven young and 25 older adults performed a Bimanual Tracking Task (BTT) during MRS acquisition. Results showed that young adults were more successful in performing the BTT than older adults (p < 0.001). Older adults revealed lower levels of both GABA+ (p < 0.05) and Glx (p < 0.001) than young adults during rest and task, but the GABA+/Glx ratio was not affected by age (p > 0.05). Also, we found no significant differences in neurometabolites between rest and task, hence no task-related modulations. A lower GABA+/Glx ratio (p < 0.05) during the task predicted better bimanual performance in older adults, whereas GABA+ and Glx at rest or during the task did not show a significant relationship with task performance (p > 0.05). These findings suggest that although neurometabolite concentrations decline with age, the relative inhibitory–excitatory balance in SMA may be preserved. Furthermore, a lower inhibitory tone appears to be critical for successful bimanual coordination in older adults.

## 1 Introduction

The performance of various simple and complex movements governs our everyday life. If impaired to a certain degree, it can negatively impact quality of life. Aging contributes to the natural decay of motor functions over time, such as in the context of bimanual coordination (Maes et al., 2017; Serbruyns et al., 2015). Hence, daily activities in which both hands are required to operate simultaneously (cooking, tying shoelaces, driving, etc.) can become more difficult to perform successfully as we age.

The Supplementary Motor Area (SMA) is one of multiple brain regions involved in motor control. It was found to play an essential role in bimanual coordination (Steyvers et al., 2003; Swinnen, 2002; Swinnen et al., 2004) due to its function of integrating interhemispheric motor signals and synchronizing movements of both limbs (Brinkman et al., 1979; Toyokura et al., 2002). In contrast to other motor regions such as the primary motor cortex (M1), SMA is involved in higher-order motor functions, including self-initiated movements or voluntary action, action–sequence learning, and conflict resolution during response planning (Debaere et al., 2004; Nachev et al., 2008; Wenderoth et al., 2005). Evidence supporting the crucial role of SMA in bimanual movements comes from studies using transcranial magnetic stimulation (TMS) and functional magnetic resonance imaging (fMRI). By temporarily disrupting the activation of SMA via repetitive TMS (rTMS), the coordination of motor planning for complex bimanual tasks was found to be impaired (Obhi et al., 2002; Serrien et al., 2002; Steyvers et al., 2003). Furthermore, fMRI studies have supported the involvement of SMA in interhemispheric coupling and sensory feedback during complex bimanual movement preparation (Karabanov et al., 2023; Welniarz et al., 2019).

Neurophysiological processes underlying efficient motor control depend on the balance between excitatory and inhibitory synaptic communication, which optimizes information processing and neural tuning in the brain (Eisenstein et al., 2024; Sohal et al., 2019). This balance, primarily guided by the key neurotransmitters gamma-aminobutyric acid (GABA) and glutamate, can affect successful motor execution and learning (Stagg et al., 2011; Vescovo et al., 2024). GABA acts as the main inhibitory neurotransmitter of the human brain, whereas glutamate serves as the principal excitatory counterpart. Edited magnetic resonance spectroscopy (MRS) at 3 Tesla enables in vivo quantification of these neurotransmitters in form of GABA+, a composite signal of GABA and additional macromolecules, and Glx, which encompasses both glutamate and glutamine. These measurements can offer relevant insights into the excitatory–inhibitory balance during complex motor control and possible age-related alterations.

Research has revealed age-related declines in both GABA+ and Glx levels in motor regions such as the primary sensorimotor cortex (SM1) and pre-SMA, which were associated with diminished motor functions in bimanual coordination and reactive motor inhibition in older adults, respectively (Hermans et al., 2018; Levin et al., 2019; Liu et al., 2024; Maes et al., 2021). Resting-state GABA+ and Glx concentrations have been shown to correlate with motor performance across different regions and task paradigms, with the SM1 being the most targeted region of interest (ROI) (Li et al., 2022).

Despite the central role of SMA in bimanual coordination, there is a lack of research exploring neurometabolites in SMA in the context of motor control and healthy aging. A study by Boy et al. (2010) found a positive relationship between SMA GABA+ levels and subconscious motor control in younger adults, which referred to automatic inhibitory mechanisms of motor control in a masked priming task. In addition, Andrushko et al. (2023) investigated GABA+ levels in SMA and SM1 before and after a handgrip-force matching task and a button-press response-time task. They found no significant task-related change in GABA+ levels at group level but associations between decreased GABA+ levels in left SM1, bilateral SMA, and behavioral performance in the contralateral hand at individual subject level (Andrushko et al., 2023). Whether these relationships are affected by age remains elusive.

To date, only one study has investigated GABA+ levels in the SMA in the context of healthy aging and bimanual coordination (Maes et al., 2021). This study confirmed an age-related decline in GABA+ levels of bilateral SM1 but reported no significant difference between young and older adults in GABA+ levels of SMA. Interestingly, SMA displayed the highest concentration of GABA+ compared with other ROIs in the motor network (Maes et al., 2021). Moreover, the study demonstrated that associations between bimanual performance and GABA+ levels were task specific and age specific. Nevertheless, the relationship between behavioral performance and GABA+ levels was observed across all ROIs, indicating that it was not region dependent (Maes et al., 2021).

Further investigation of the neurochemical inhibitory–excitatory balance in SMA might greatly impact the understanding of its contribution to motor control in aging given its high GABA+ concentration and yet unspecified relationship with bimanual coordination. Similar to the aforementioned study, the majority of previous MRS research has investigated resting-state neurometabolite concentrations, which do not capture dynamic chemical changes in response to an active motor task. Functional MRS (fMRS) addresses this limitation by measuring dynamic changes of GABA+ and Glx in direct response to task-specific demands (Li et al., 2024).

A recent meta-analysis of fMRS studies revealed heterogeneous findings across paradigms, domains, and ROIs regarding task-related GABA+ and Glx modulations. Nevertheless, they reported a general trend toward task-induced increases in Glx levels and decreases in GABA+ levels in previous studies (Pasanta et al., 2023). While task-induced changes across studies in Glx were found to be significant, they exhibited only small to moderate effect sizes (Pasanta et al., 2023). Additional studies support training-induced declines in GABA+ concentrations across multiple motor learning paradigms (Li et al., 2024; Maes et al., 2022). Research about task-induced modulations in aging adults is rather limited and potentially mediated by lower baseline GABA+ and Glx levels in older adults (King et al., 2020). Yet, transient GABA+ changes have been reported in SM1 in both young and older adults during an action-selection task (Maes et al., 2022). Although older adults exhibited lower GABA+ levels than young adults, a greater task-induced decrease in GABA+ was linked to better bimanual performance, suggesting effective modulatory mechanisms. In contrast, young adults demonstrated an inverse relationship, indicating that greater task-induced modulation was associated with poorer performance (Maes et al., 2022).

To the best of our knowledge, no study has examined motor task-induced transient modulations of GABA+ and Glx in the SMA in healthy aging. Since SMA is assumed to play a key role in the network orchestrating complex motor functions, further investigation of inhibitory–excitatory mechanisms would help to understand the role of SMA in bimanual coordination and how it potentially impacts age-related decline in motor functions.

The aim of this study is to investigate age-related dynamic neurometabolite modulations in the SMA during bimanual coordination. We examined GABA+ and Glx levels—as well as their relative ratio (GABA+/Glx)—in younger and older adults and their dynamic changes during a bimanual coordination task in contrast to baseline (rest). In addition, we explored the relationship between resting and task-related GABA+ or Glx levels and bimanual performance in younger and older adults. We hypothesized (1) that baseline GABA+ and Glx levels will be lower in older than in younger adults and (2) that bimanual task performance will induce a task-related decrease in GABA+ levels and a task-related increase in Glx. Finally, we explored how the GABA+/Glx ratio is affected by aging in the context of bimanual coordination.

## 2 Methodology

### 2.1 Participants

A total of 52 participants were recruited including 27 younger (age = 25.3 ± 4.8 [mean ± SD] years, range = 20–39 years; 15 females) and 25 older (age = 68.4 ± 4.7 [mean ± SD] years, range = 61–79 years; 15 females) healthy adults. All participants were right-handed, non-smokers with normal or corrected-to-normal vision. Participants were excluded if they reported a history of major neurological or psychiatric disease, showed MR-related contraindications, were taking recreational drugs or reported a history of drug abuse, were chronically using medication interfering with the central nervous system, were physically not able to perform the motor task, or had a history of high bimanual coordinative demands (gaming, playing a musical instrument, etc.) within the past 5 years. The Montreal Cognitive Assessment (MoCA) (Nasreddine et al., 2005) as administered to screen for cognitive impairments (mean score = 28.10, range = 26-30, exclusion < 26) and right handedness was assessed using the Edinburgh Handedness questionnaire (Oldfield, 1971). All participants provided full written informed consent before participation. All methods were carried out in accordance with the Declaration of Helsinki. Note that this study was not pre-registered before data collection. The study was approved by the local ethics committee of UZ/KU Leuven (reference S66028).

### 2.2 Procedure

**Fig. 1.**
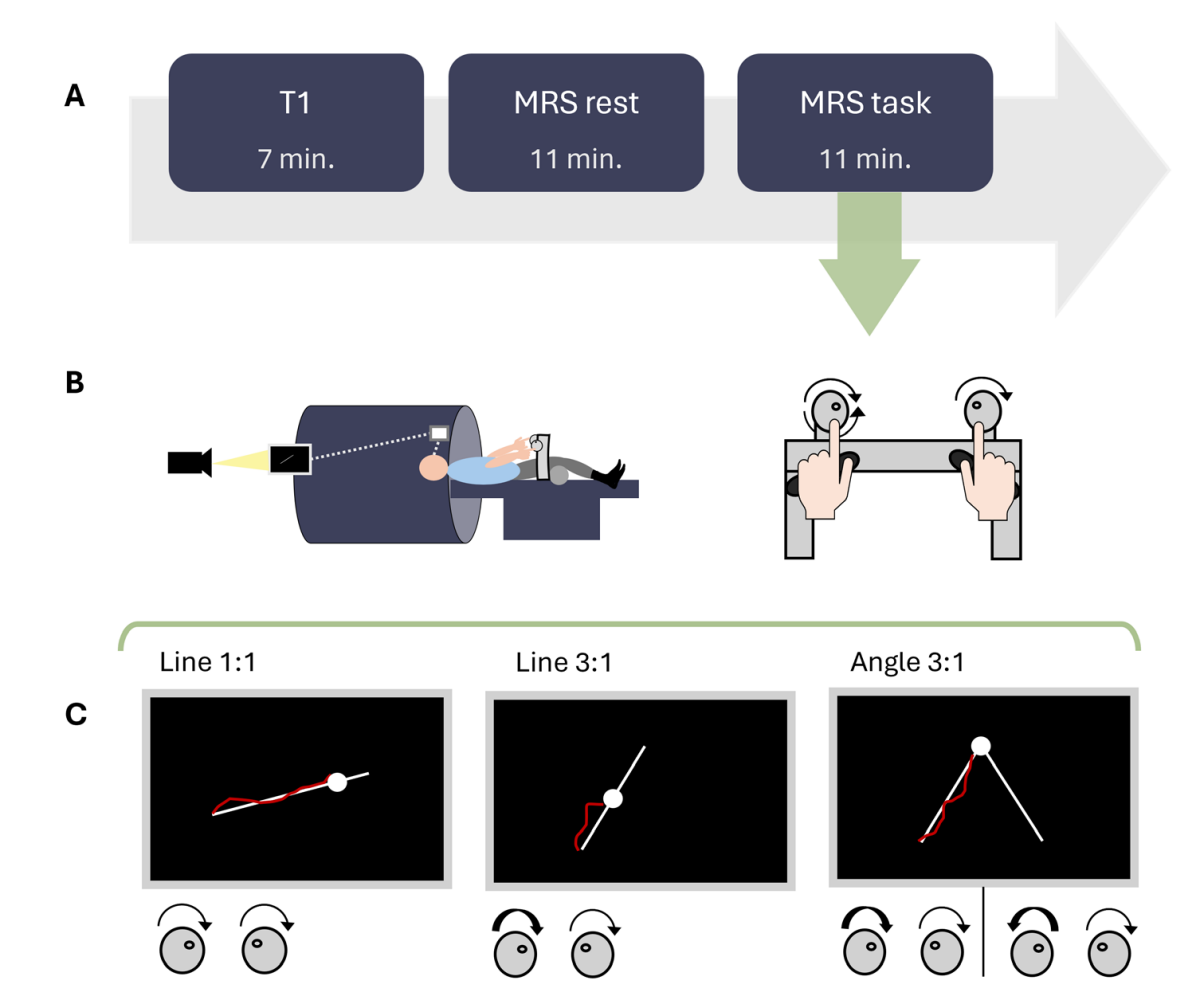
(A) Overview of the MRS experiment. Note that in addition a FLAIR was acquired after the T1 and an fMRI was acquired after MRS, which are not displayed in this figure. (B) Bimanual Tracking Task (BTT) setup in the MRI scanner. (C) Behavioral task conditions with their respective hand movement directions (clockwise or counter-clockwise) and frequency ratios (1:1 or 3:1 left:right hand). White target figures are shown for each condition including the white target dot (moving cursor) and red feedback line of movement trajectory.

In the first experimental session, questionnaires were administered, and participants underwent a familiarization of the Bimanual Tracking Task (BTT) in a mock-scanner environment until reaching a score of at least 10% (total performance scores ranging from 0 to 100%; for a detailed description of the scoring method, see Supplement 1). On the day of the MRI scan, participants were asked to abstain from caffeine and alcohol consumption. When participants were placed in the scanner, the BTT setup was installed above their hips to perform the task subsequently during the MRS and fMRI sequences, lying supine in the scanner. An overview of the MRI protocol is visualized in Figure 1A. First, a T1-weighted (~7 min) and FLAIR image (not shown in Fig. 1A, ~4 min) were acquired, followed by the first MRS acquisition (resting state, ~11 min), in which participants visually focused on a white cross on a screen, which was projected onto and viewed via a mirror (Fig. 1B). Afterward, a second MRS acquisition (task related, ~11 min) was acquired while participants were performing the BTT (Fig. 1C). Lastly, after MRS, participants performed the task during an fMRI scan (not shown in Fig. 1A, ~6 min). Here, we present the analysis and results obtained from the data acquired during resting-state and task-related MRS.

### 2.3 Imaging data acquisition

Neuroimaging data were acquired at the University Hospital Leuven using a Philips Achieva dStream 3 Tesla scanner with a 32-channel head coil (Philips; Best, the Netherlands). A high-resolution three-dimensional T1-weighted image (MPRAGE, echo time [TE] = 2.5 ms, repetition time [TR] = 5.8 ms, flip angle 8°, field of view = 256 x 240 x 166 mm, 208 transverse slices, voxel size = 0.8 x 0.8 x 0.8 mm, acquisition time ~7 min) was acquired for MRS voxel placement and tissue correction during MRS data processing.

**Fig. 2.**
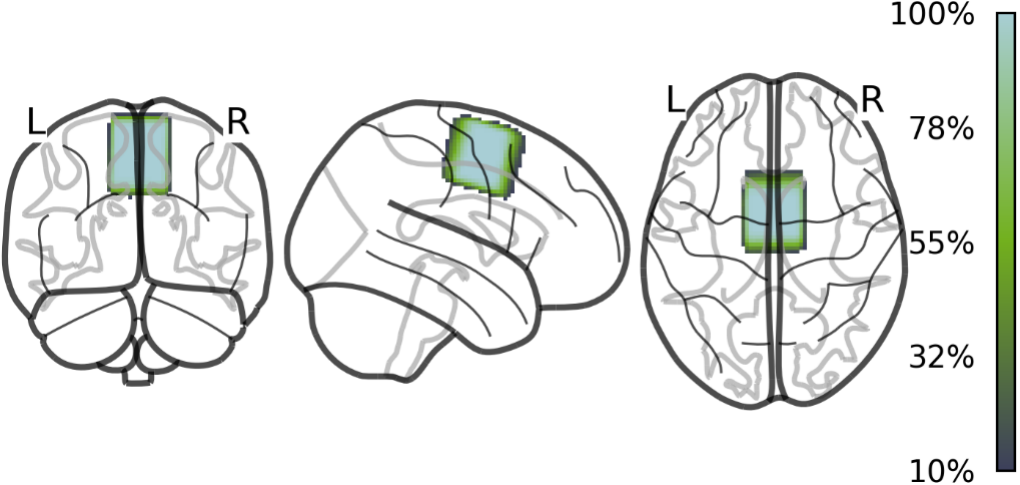
Voxel placement and percentage of voxel overlap in SMA across all participants.

A Hadamard Encoding and Reconstruction of MEGA-Edited Spectroscopy (HERMES) sequence (Saleh et al., 2016) was used for MRS acquisition during rest and task (320 water-suppressed and 16 water-unsuppressed averages, acquisition time of 11 min per block, MOIST water suppression, 2000 Hz spectral width, 1024 datapoints, first-order PB-shimming), which allows for the quantification of GABA+, Glx, and glutathione (GSH) with editing pulses (basing pulse duration = 20 ms) being applied at 1.9 ppm (GABA-ON) and 4.56 ppm (GSH-ON) using 4 Hadamar-encoded sub experiments (Saleh et al., 2016). Note that GSH data have not been analyzed in the context of this study. Two identical blocks of MRS (resting-state vs. task-related) were acquired to assess neurometabolite levels in bilateral SMA with a 3 x 3 x 3 cm^3^ voxel. Voxel placement was done by first drawing a horizontal line from the anterior commissure (AC) to the posterior commissure (PC) in the sagittal view (AC-PC line). Two lines were drawn perpendicular to this AC-PC line at the height of the AC and PC. The voxel was placed between the two perpendicular lines along the cortical surface in the sagittal plane. The top of the voxel was rotated to be parallel to the cortical surface in sagittal and coronal views and placed at the midline of the brain in the transverse view to cover left and right SMA (Biria et al., 2023; Maes et al., 2021). Figure 2 illustrates voxel placement and overlap across all participants (Truong et al., 2020).

### 2.4 Behavioral task

The BTT, a bimanual visuomotor tracking task (Sisti et al., 2011), was optimized for the MR-scanner using a non-ferromagnetic task set-up which was positioned above participant’s hips while lying supine in the scanner (Fig. 1B) to assess bimanual coordination. Participants were instructed to rotate two dials (one per index finger) to follow a white moving cursor along a target line on the screen. While lying supine in the scanner with the BTT set-up above their hips, the task was visualized via a mirror and projector in the MR scanner allowing them to lay still despite their hand movements. Each trial of the BTT started with the presentation of the target line appearing on a black background until the end of the trial. One second after the onset presentation, the target cursor, represented by a white dot, started to move along the target line at a constant speed (trial duration: 6 s). The duration of the inter-trial interval was 1 s. Participants’ bimanual dial rotations were visualized by a red line providing concurrent feedback of their actual movement trajectory. Three separate conditions (Line 1:1, Line 3:1, Angle 3:1) required cyclical movement at various inter-hand frequency ratios in a clockwise or anticlockwise direction. Frequency ratio of 1:1 required participants to move both hands at the same speed in a clockwise direction (Line 1:1), whereas 3:1 frequency ratio required the left hand to move three times faster than the right hand (Fig. 1B) in a clockwise direction (Line 3:1) or both clockwise and anti-clockwise directions (Angle 3:1). Participants’ performance was quantified as a percentage score (0–100%) per trial based on the minimum Euclidean distance to the target line, speed, and movement direction (Adab et al., 2020; Hehl et al., 2024). A detailed description of the scoring method is provided in the Supplementary Materials (Supplement 1). For further analysis, the mean score across all conditions was used as the final BTT score.

### 2.5 MRS data analysis

Data were preprocessed and quantified using Gannet v3.3.2 (Edden et al., 2014). The preprocessing steps included zero-filling and the application of a 3 Hz exponential line broadening filter. Multi-step frequency and phase correction (FPC) were performed using spectral registration for HERMES (Mikkelsen, Saleh, et al., 2018). Difference spectra were fitted within the 2.79 to 4.10 ppm range to quantify the GABA+ (3.0 ppm) and Glx (3.75 ppm) peaks using a three-Gaussian function with least-squares fitting. Metabolite concentrations were referenced to the water signal using a Gaussian–Lorentzian model between 3.8 and 5.6 ppm on the water-unsuppressed spectrum. Structural T1-weighted images were segmented to identify gray matter (GM), white matter (WM), and cerebrospinal fluid (CSF) using SPM12 (Statistical Parametric Mapping, Wellcome Trust Centre for Human Neuroimaging, University College; London,UK), and each MRS voxel was co-registered to its respective anatomical image. Metabolite levels were tissue corrected using group-normalized alpha correction (Harris, Puts, & Edden, 2015; Porges et al., 2017).

**Fig. 3.**
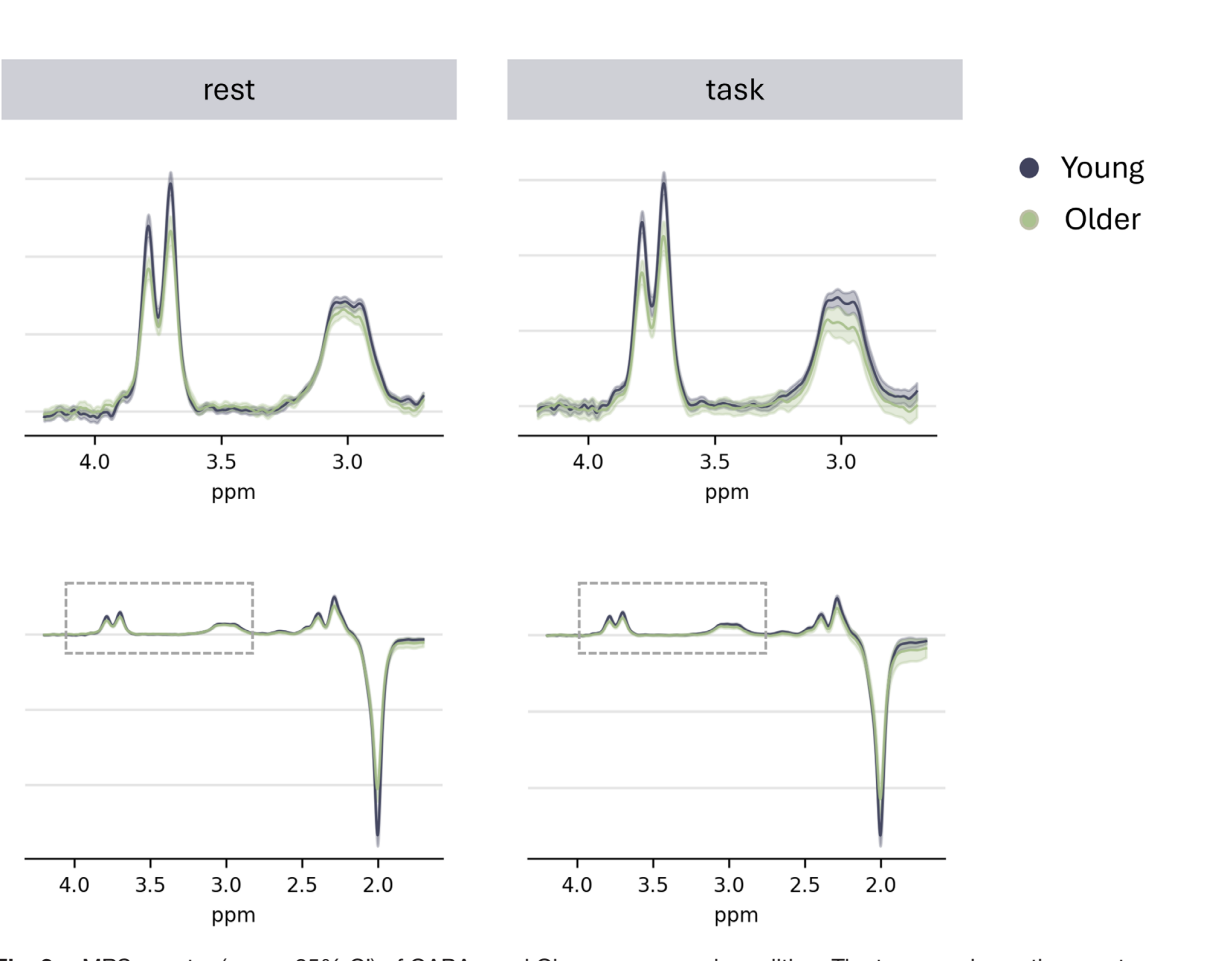
MRS spectra (mean; 95% CI) of GABA+ and Glx per group and condition. The top row shows the spectra on a reduced ppm scale, highlighted by the dotted box in the full ppm scale presented in the bottom row.

All processed data were visually inspected for artifacts and overall spectral quality. Additionally, quantitative data quality measures for GABA+ and Glx were based on fitting errors, with a cutoff of >12% (Puts et al., 2018), signal-to-noise ratio (SNR), and full-width-half-maximum (FWHM). Data were excluded if their SNR was below 3 standard deviations (SD) from the mean, or if their FWHM exceeded 3 SD above the mean. One participant showed a high GABA+ fit error (~19%) in the task condition spectra. Visual inspection yielded that the spectral alignment algorithm was introducing this error, which could not be avoided using other alignment algorithms. Without spectral alignment, the data showed clear visual peaks of Glx and GABA, and quality measures (fit error, SNR, and FWHM) and were within the specified limits. Hence, resting- and task-related data were included for analysis for this participant without applying any spectral alignment. Data from two participants were excluded due to poor spectral quality in the resting-state condition (older adults: n = 2, based on fit error > 12%). An overview of data quality measures for GABA+ and Glx for each age group and condition is given in Table 1 and Supplement 2. MRS spectra are visualized in Figure 3. To assess inhibitory–excitatory balance, we additionally calculated the ratio of GABA+ and Glx, hereafter referred to as GABA+/Glx.

**Table 1.** MRS data quality metrics and tissue compositions.

			GABA+			Glx		
Metrics	Condition		YA	OA	p	YA	OA	p
Fit error (%)	Rest		5.17 ± 1.12	∇ 5.14 ± 0.94	0.799	2.62 ± 0.63	3.11 ± 0.75	***0.009***
	Task		∇ 5.21 ± 1.66	∇ 5.67 ± 1.73	0.167	∇ 2.64 ± 0.55	∇ 3.16 ± 1.11	***0.023***
		p	0.469	0.142		0.982	0.901	
								
SNR	Rest		14.00 ± 2.25	11.83 ± 2.17	***0.001***	∇ 24.71 ± 5.92	∇ 17.11 ± 2.72	***<0.001***
	Task		13.88 ± 2.71	11.48 ± 1.84	***<0.001***	23.39 ± 4.14	18.11 ± 3.50	***<0.001***
		p	0.828	0.487		0.524	0.419	
								
FWHM (Hz)	Rest		23.86 ± 1.97	22.97 ± 2.11	0.126	∇ 12.54 ± 0.89	∇ 13.20 ± 1.80	***0.007***
	Task		23.30 ± 1.78	22.8 ± 2.02	0.354	∇ 12.80 ± 1.70	∇ 13.32 ± 1.42	0.488
		p	0.177	0.987		0.141	0.917	
								
Voxel fraction								
Gray matter			0.54 ± 0.05	0.47 ± 0.03	***<0.001***			
White matter			0.34 ± 0.04	0.33 ± 0.05	0.094			
Cerebrospinal fluid			∇ 0.12 ± 0.04	∇ 0.20 ± 0.08	***<0.001***			

Data are presented as mean ± SD for parametric tests and median ± IQR for non-parametric tests (∇).

Group comparisons used two-sample t-tests or Wilcoxon rank-sum test, while comparisons between rest and task conditions used paired-sample t-tests or Wilcoxon signed-rank test. Bold numbers indicate statistically significant differences (p < 0.05) between groups or between conditions. Quality metrics were obtained from Gannet.

FWHM = full-width-half-maximum; SNR = signal-to-noise ratio; YA = young adults; OA = older adults.

Additionally, metabolite concentrations of Glx (and the ratio of GABA+/Glx) were quantified using Osprey (Oeltzschner et al., 2020), due to its more reliable performance in metabolite estimation using a linear combination modeling (LCM) approach (Craven et al., 2022; Song et al., 2022; Zöllner et al., 2022). Analysis steps included eddy-current correction using the water reference, frequency, and phase correction via robust spectral registration (Mikkelsen et al., 2020) and residual water filtering. Reconstructed spectra were fitted using Osprey LCM with a metabolite fitting range of 0.5–4.0 ppm and baseline knot spacing at 0.4 ppm. Glx (GABA+/Glx) was quantified using group-normalized alpha correction. The subsequent analyses use GABA+ levels quantified with Gannet and Glx levels, as well as GABA+/Glx ratio, quantified with Osprey.

### 2.6 Statistical analysis

Statistical analyses were performed in R (Version 4.4.2) and RStudio using packages “lme4”, “car”, “performance” (Bates et al., 2008; Fox et al., 2018; Lüdecke et al., 2021). The significance level was set at α = 0.05, unless otherwise specified. Stepwise backward modeling was applied for all models, in which interactions and/or factors not reaching statistical significance were removed. Normality of residuals was assessed by Q-Q plots and Shapiro–Wilk tests.

To assess task-related changes in GABA+ and Glx levels in young and older adults, three linear mixed models were constructed with NEUROMETABOLITE levels (GABA+ vs. GLX vs. GABA+/GLX) as the dependent variable and GROUP (young vs. older), CONDITION (rest vs. task) as fixed factors and GROUP x CONDITION interaction. WATER_FWHM was included in all models as a covariate to control for task-related BOLD effects (Craven, Bell, et al., 2024; Stanley et al., 2018). PARTICIPANT was included as a random intercept.

Differences between groups in behavioral performance of the BTT task were assessed via independent samples t-test using BTT SCORE as the dependent variable and GROUP (young vs. older) as independent variable.

Multiple linear regression was implemented to investigate the relationship between bimanual performance and neurometabolites (GABA+ vs. GLX vs. GABA+/GLX) across groups. Four models were constructed to predict performance including either resting-state or task-related neurometabolite levels or ratios. In the first two models, based on either rest or task-related neurometabolites, BTT SCORE was included as the dependent variable, while GROUP (young vs. older), GABA+, GLX, and their interactions with GROUP (i.e., GROUP x GABA+ and GROUP x GLX) were implemented as predictors. Two additional models were constructed to predict behavioral performance based on the GABA+/Glx ratio for either rest or task. Similarly, BTT SCORE was implemented as the dependent variable with GROUP (young vs. older), GABA+/GLX and its interaction with GROUP (i.e., GROUP x GABA+/GLX) as predictors. In all models including task-related metabolites, WATER_FWHM was added as a covariate of no interest to control for task-related BOLD effects (Craven, Bell, et al., 2024).

## 3 Results

### 3.1 Neurometabolite modulations in young and older adults

**Fig. 4.**
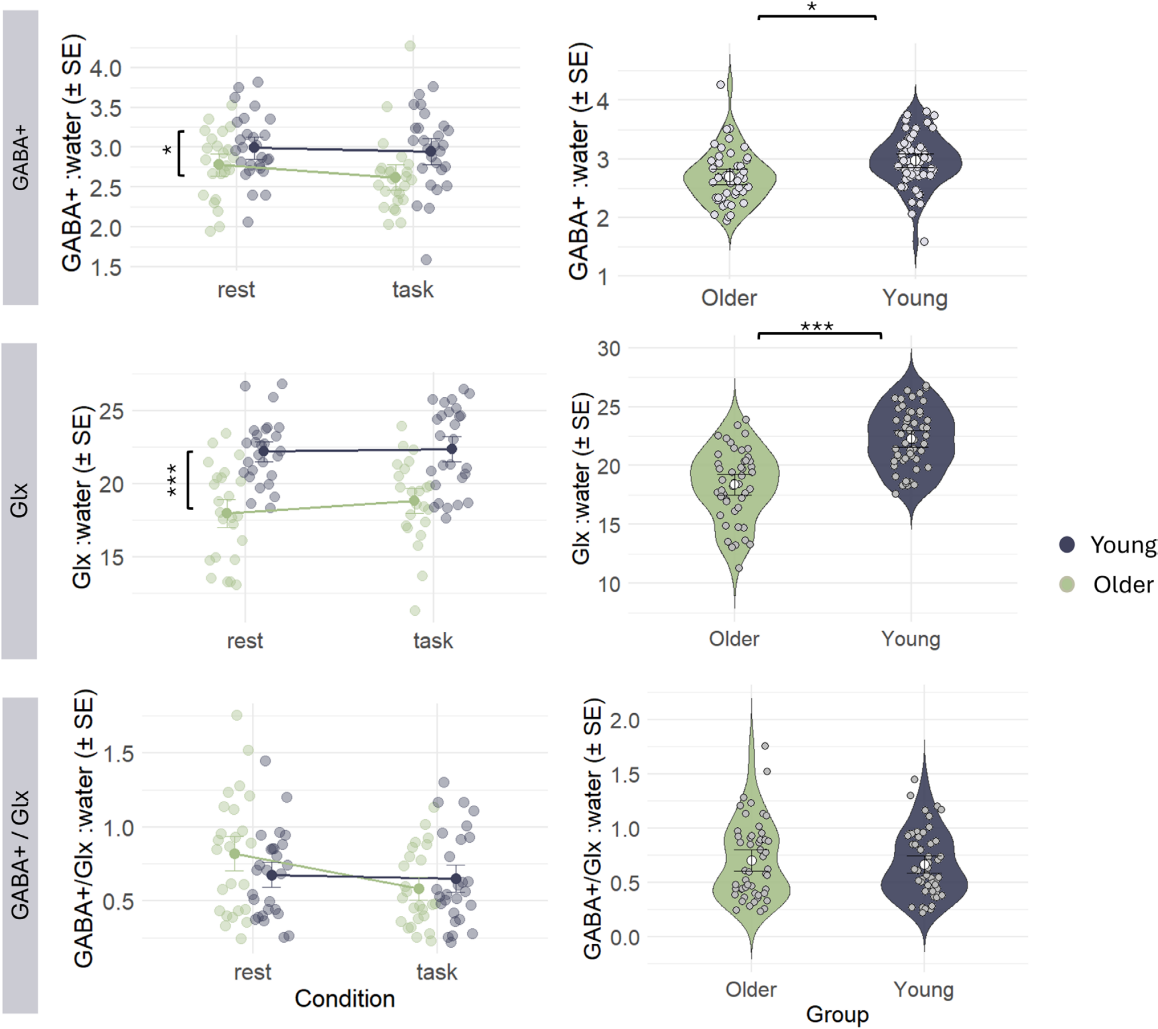
Mean (SE) of neurometabolite concentrations of GABA+, Glx, and GABA+/Glx ratio per group (young vs. older) and condition (rest vs. task). Pooled data (mean± SE) of both conditions are shown on the right (violin plots) to visualize age group effects. Metabolite levels were tissue corrected using group-normalized alpha correction. GABA+ levels were quantified using Gannet. Glx and GABA+/Glx were quantified using Osprey.

Mean values of GABA+ and Glx concentrations in young and older adults per condition are depicted in Figure 4. Model output tables are given in the Supplementary Materials (Supplement 3).

#### 3.1.1 Age-related differences in GABA+, but no task-related changes

Results of the linear mixed model including GABA+ as outcome variable revealed no significant CONDITION x GROUP interaction (F_1,48.5_ = 0.44, p = 0.512) and no significant effect of CONDITION (F_1,49.2_ = 1.61, p = 0.210). After stepwise removal of each of these terms, a significant effect of GROUP (F_1,47.99_ = 6.51, p = 0.014; β [young] = 0.274) remained in the final model indicating lower GABA+ levels in the older adult group.

#### 3.1.2 Age-related differences in Glx, but no task-related changes

Results of the linear mixed model including GLX as outcome variable quantified by Osprey showed no significant CONDITION x GROUP interaction (F_1,47.6_ = 0.62, p = 0.434) and no significant effect of CONDITION (F_1,50.7_ = 2.42, p = 0.126). A significant effect of GROUP (F_1,48_ = 31.07, p < 0.001; β [young] = 4.264) remained in the final model, which indicates lower Glx levels in the older adult group.

#### 3.1.3 No age-related differences and task-related changes in GABA+/Glx ratio

Results of the linear mixed model with GABA+/GLX ratio as outcome measure quantified by Osprey revealed no significant CONDITION x GROUP interaction (F_1,48.9_ = 2.96, p = 0.092) and a trending effect of CONDITION (F_1,50.6_ = 3.13, p = 0.083). Additionally, GROUP had no significant effect (F_1,49.1_ = 0.06, p = 0.810) in the final model indicating no difference in GABA+/Glx ratio between young and older adults.

### 3.2 Differences between young and older adults in bimanual coordination performance

**Fig. 5.**
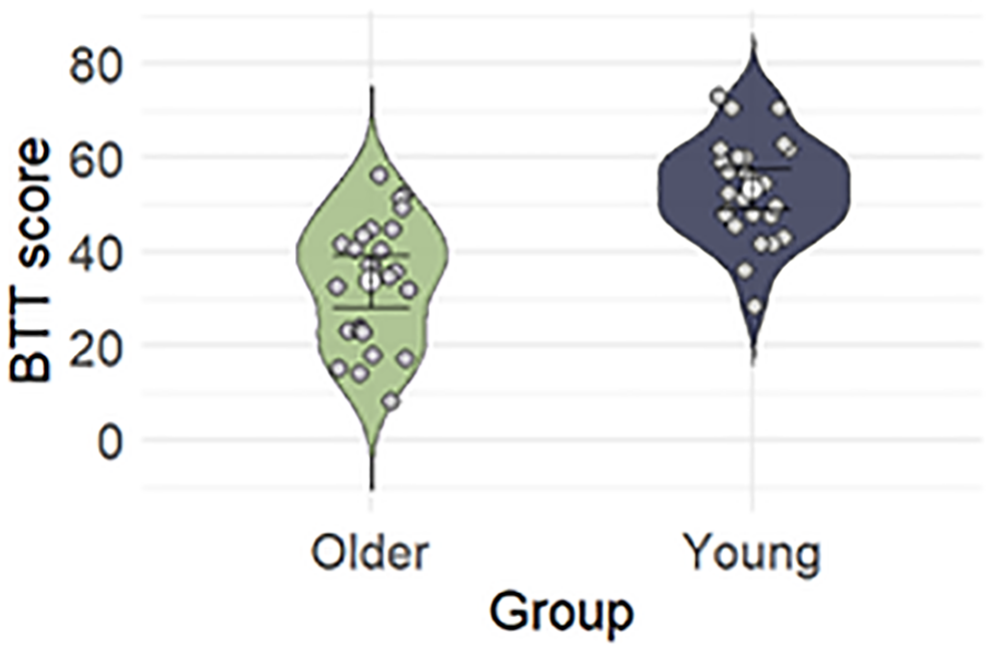
Behavioral performance (mean ± SE) of the Bimanual Tracking Task (BTT) in older adults and young adults.

Behavioral data of the BTT score in both age groups are visualized in Figure 5. Independent samples t-test revealed a significant difference between groups in BTT SCORE (t _47_ = -5.849, p-value < 0.001).

### 3.3 Relationship between bimanual coordination and neurometabolites

**Fig. 6.**
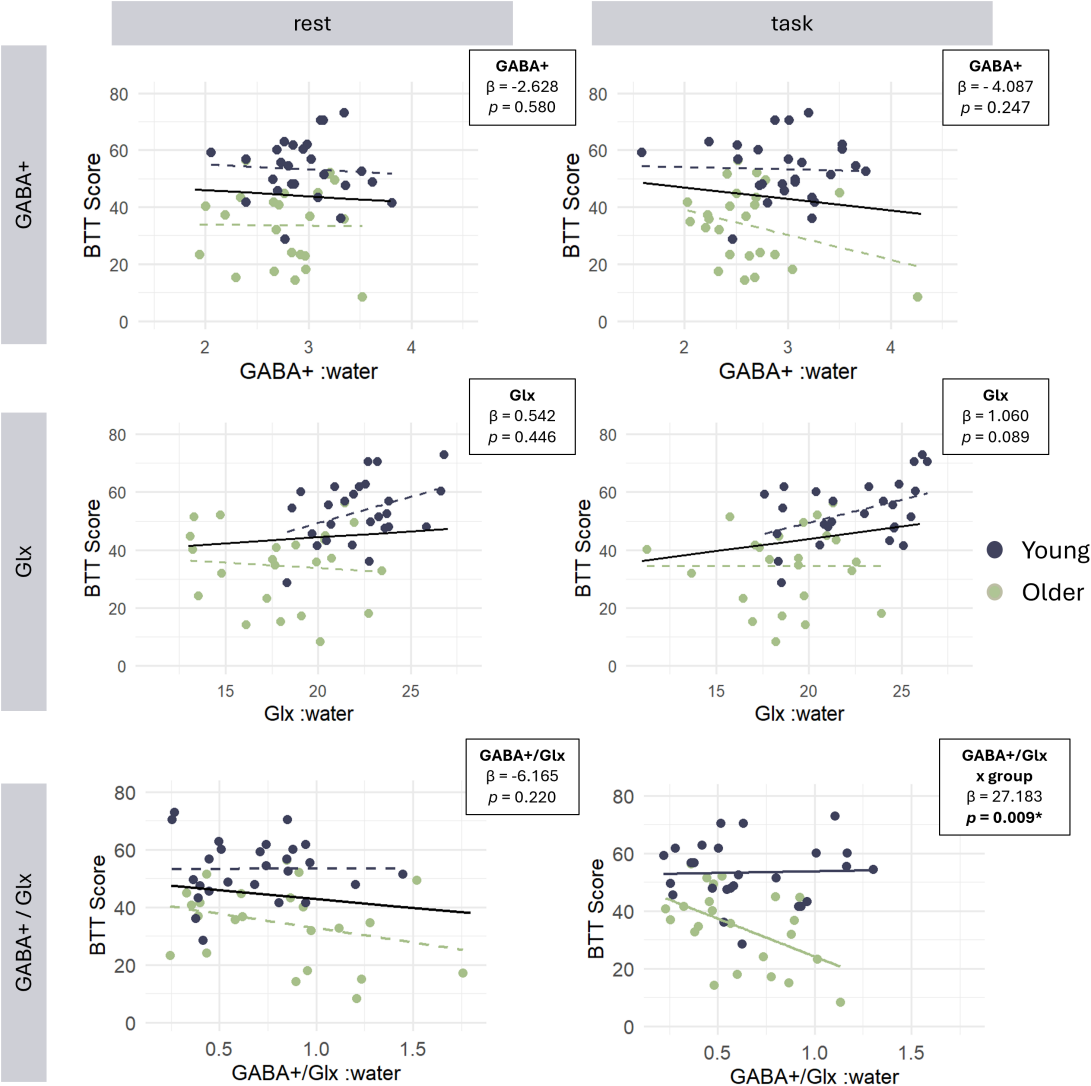
Relationship between resting-state or task-related neurometabolite concentrations (GABA+, Glx, GABA+/Glx ratio) and participants’ performance of the Bimanual Tracking Task (BTT). Slopes (β) and significance levels (p) are depicted for the main effects (solid black line) of metabolites on BTT score. Individual slopes per group are indicated by a dashed line. Interaction effects of group and metabolites on BTT were significant in GABA+/Glx task only. GABA+ levels were quantified using Gannet. Glx and GABA+/Glx were quantified using Osprey. * Indicates significant results after FDR correction.

The relationship between BTT scores and neurometabolite concentrations of GABA+, Glx, and GABA+/Glx ratio is visualized in Figure 6. Output tables of the full and final models are given in the Supplementary Materials (Supplement 4). False discovery rate (FDR) correction was applied to the effects’ p-values of the final models using the Benjamini–Hochberg (BH) procedure (Benjamini et al., 1995) with q < 0.05. Prediction of BTT performance based on resting-state GABA+ and Glx levels

#### 3.3.1 Prediction of BTT performance based on resting-state GABA+ and Glx levels

After the removal of non-significant interaction terms GROUP x GLX (t = 1.59, p = 0.121, β = 2.276) and GROUP x GABA+ (t = -0.13, p = 0.895, β = -1.253) from the linear regression model, neurometabolites showed no significant predictors for BTT SCORE based on GABA+ (t = -0.58, p = 0.580, β = -2.628) and GLX (t = 0.77, p = 0.446, β = 0.542). A significant effect of GROUP (t = 5.44, p < 0.001, β = 18.777) remained in the final model, indicating an age-related difference (see Section 3.2) in motor performance. After FDR correction, a significant effect of GROUP (q < 0.001) remained in the model.

#### 3.3.2 Prediction of BTT performance based on task-related GABA+ and Glx levels

Results of the multiple linear regression analysis showed no significant interactions of GROUP x GLX (t = 1.30, p = 0.200, β = 1.597) and GROUP x GABA+ (t = 0.726, p = 0.472, β = 5.362). No significant effect of GLX (t = 1.74, p = 0.089, β = 1.060) and GABA+ (t = -1.70, p = 0.097, β = -6.241) and a significant effect of GROUP (t = 3.40, p < 0.001, β = 17.443) on BTT performance remained, again indicating an age-related difference in motor performance (see Sections 3.2, 3.3.1).

#### 3.3.3 Prediction of BTT performance based on resting-state GABA+/Glx ratio

Multiple linear regression analysis revealed no significant effects of GABA+/GLX_REST x GROUP (t = 0.99, p = 0.329, β = 10.098) and GABA+/GLX_REST (t = -1,243, p = 0.220, β = -6.165) on BTT SCORE. Upon stepwise removal of these terms, a significant effect of GROUP (t = 5.44, p < 0.001, β = 18.777) remained in the model following FDR correction (q < 0.001). These results suggest that there is no relationship between resting-state GABA+/Glx and BTT performance. These results replicate the above-mentioned age-related difference in motor performance (see Sections 3.2, 3.3.1, 3.3.2), which is not affected by GABA+/Glx ratio during rest.

#### 3.3.4 Prediction of BTT performance based on task-related GABA+/Glx ratio

The results of the task-related model showed that there was a significant interaction term of GABA+/GLX_TASK x GROUP (t = 2.26, p = 0.029, β = 27.183) in addition to a significant effect of GABA+/GLX_TASK (t = -2.75, p = 0.009, β = -26.182). But there was no significant effect of GROUP (t = 0.30, p = 0.767, β = 2.512). Effects remained significant following FDR correction for GABA+/GLX_TASK (q = 0.012) and GABA+/GLX_TASK x GROUP interaction (q = 0.029). This indicates that GABA+/Glx ratio during task execution affects BTT performance differently depending on age group.

Post hoc simple effects analysis revealed that there was no significant relationship between GABA+/GLX_TASK and BTT performance in young adults (t = 0.17, p = 0.870, β = 1.138), but a significant negative relationship between GABA+/GLX_TASK and BTT performance in older adults (t = -2.66, p = 0.015, β = -26.259).

## 4 Discussion

The aim of this study was to examine neurochemical differences in the SMA between both age groups at rest and during performance of a bimanual coordination task. We investigated inhibitory–excitatory mechanisms, in form of GABA- and glutamatergic modulations, underlying bimanual motor control in young and older adults and explored the relationship between behavioral performance and neurometabolite concentrations.

Several key findings became evident. First, we found an age-related decrease in GABA+ and Glx levels in SMA. Second, there were no task-related modulations in either GABA+, Glx, or GABA+/Glx ratio in response to the bimanual motor task. Third, performance on the BTT was dependent on age group, with young adults outperforming older adults. Fourth, there was no relationship between baseline neurometabolite levels in the SMA and BTT performance. Yet, a lower ratio of GABA+/Glx predicted better bimanual performance in older adults, while no significant relationship existed between task-based GABA+ or Glx levels and BTT performance.

### 4.1 Age-related difference in GABA+ and Glx in the absence of task-related modulation

Our results demonstrated lower GABA+ and Glx concentrations in older than in young adults, which is in agreement with previous studies that revealed decreased concentrations of GABA+ and Glx in multiple ROIs of the motor network (Hermans et al., 2018; Levin et al., 2019; Maes et al., 2021; Rodríguez-Nieto et al., 2025). Advanced analysis using Osprey LCM for the quantification of Glx confirmed an age-related decline in our study. This decline aligns with age-related depletion of glutamatergic and GABAergic neurons observed in animal studies (Heumann et al., 1983; Rozycka et al., 2017). However, our findings are in contrast with those of Maes et al. (2021), who did not reveal significant age-related differences in GABA+ concentrations of SMA. Instead, they reported lower GABA+ levels in older adults than in young adults only for the SM1. Although both studies used the same SMA voxel definition, Maes et al. (2021) quantified GABA+ using the MEGA-PRESS sequence, whereas we implemented HERMES. It is relevant to note that perhaps their study design, which included seven ROIs and three task paradigms, may have reduced statistical power to detect subtle regional changes (Maes et al., 2021). Since research about SMA-related differences in neurometabolites in the context of aging and motor control is still scarce, conclusions should be drawn with caution. Nevertheless, we provide first evidence for an age-related decline in GABA+ and Glx in the SMA, consistent with findings in other brain regions (Gao et al., 2013; Roalf et al., 2020).

Less research has been done on dynamic GABA- and glutamatergic modulations in response to a bimanual motor task, and so far, no study has investigated task-related neurometabolite changes in the SMA. Our results did not confirm our hypothesis, as there were no significant changes in neurometabolites between the rest and task conditions. This is in contrast to previous research, which showed that SM1 GABA+ levels of both young and older adults decreased during bimanual motor task execution (Maes et al., 2022). Overall, studies using fMRS investigating GABAergic and glutamatergic modulations show low effect sizes and large heterogeneity of study designs. Yet, most studies reported a positive effect for Glx levels and only trending negative effects for GABA+ levels in response to a task, for a review see Pasanta et al. (2023). These effects, along with the observed heterogeneity, may be further influenced by brain region-specific variations in Glx and GABA+ as well as age-related neurometabolic changes, potentially contributing to the contrasting findings in our study.

Since this is the first study to examine motor task-related modulations in the SMA, any conclusions about its modulatory role in bimanual coordination remain speculative. Based on the non-significant effect in our study, it is possible that the SMA plays a less prominent role in BTT performance compared with other regions, such as SM1, resulting in weaker or no modulation of GABAergic and glutamatergic mechanisms. Notably, we found no difference in BOLD effects between rest and task conditions (see FWHM water, Table 2, Supplement 5 Fig. S1). This finding aligns with the lack of task-related modulation in neurometabolites and may suggest that activation changes in the SMA play a limited role under the present task demands (Just, 2024; Zhu et al., 2001). Perhaps task modulations in SMA are influenced by higher spatiotemporal complexity levels (Debaere et al., 2004) which might not have been sufficiently challenging in our current study. To further explore the modulatory role of SMA in bimanual coordination, future studies may consider differentiating between levels of task demands using event-related designs or advanced techniques such as concurrent fMRI-fMRS (Craven, Dwyer, et al., 2024).

**Table 2.** Extended MRS data quality of water and N-acetyl aspartate (NAA).

			Water			NAA		
Metrics	Condition		YA	OA	p	YA	OA	p
FWHM (Hz)	Rest		∇ 8.79 ± 0.49	∇ 8.30 ± 1.22	***0.022***	∇ 8.15 ± 1.22	∇ 8.91 ± 1.20	***0.004***
	Task		∇ 8.91 ± 0.85	∇ 8.18 ± 1.28	***0.012***	∇ 8.62 ± 1.40	∇ 8.78 ± 1.32	0.138
		p	0.336	0.704		0.336	0.704	
							
Frequency offset (ppm)	Rest		∇ 0.0214 ± 0.0097	∇ 0.0274 ± 0.0092	***0.044***			
	Task		∇ 0.0202 ± 0.0092	∇ 0.0241 ± 0.0102	***0.014***			
		p	***0.008***	***0.024***				

Data are presented as median ± IQR for nonparametric tests (∇).

Group comparisons used Wilcoxon rank-sum test, while comparisons between rest and task conditions used Wilcoxon signed-rank test. Bold numbers indicate statistically significant differences (p < 0.05) between groups or between conditions. Quality metrics were obtained from Gannet.

FWHM = full-width-half-maximum; YA = young adults; OA = older adults.

### 4.2 Age- and task-related effects on the balance between GABA+ and Glx

To better understand the balance between inhibitory and excitatory mechanisms within SMA, we utilized the ratio of GABA+/Glx as an additional outcome measure in our analysis. We did not observe a significant effect of task modulation on GABA+/Glx, which is in line with the absence of task-related modulations in individual metabolites in the aforementioned results. Although SMA activation during the BTT has been consistently found using fMRI (Van Ruitenbeek et al., 2022), the modulation of inhibitory–excitatory mechanisms might not directly be affected by the current task. Potential variations focusing on more internally guided movements, known to specifically engage the SMA (Beets et al., 2015), could induce stronger modulatory effects of GABA+/Glx.

In contrast to our previous analysis of individual metabolites, GABA+/Glx ratio showed no significant effect of age group. This suggests that although both GABA+ and Glx concentrations decreased in older adults compared with young adults, their relative balance remained unchanged in our study. This finding contrasts with the expectation that the balance between inhibitory and excitatory pathways is typically altered with aging (Rozycka et al., 2017). Previous research about the inhibitory–excitatory balance in the inferior frontal cortex (IFC) and inferior parietal lobule (IPL) aligns with our results given that they reported no difference in GABA+/Glx at baseline between young and older adults (Rodríguez-Nieto et al., 2024). Interestingly, the decrease in GABA+ and Glx concentrations with aging has been found in multiple studies when analyzed separately (Hermans et al., 2018; Levin et al., 2019; Maes et al., 2021), but little research has been done to directly compare the ratio between them. However, exploring the relative balance between inhibition and excitation may be crucial since inhibitory synapses are known to regulate excitation to fine tune neural networks (Froemke, 2015). Our results suggest that while overall neurotransmitter levels decrease in aging, the balance between inhibitory–excitatory mechanisms in SMA might be preserved in older adults. This tentative conclusion warrants further investigation.

### 4.3 Associations between age group, metabolites, and bimanual coordination

Our results have shown that age is a significant predictor of bimanual performance. As expected and in agreement with previous research, we found that young adults performed overall better on the BTT than older adults (King et al., 2018; Maes et al., 2017). Due to the complex nature of the task, it is not surprising that older adults performed the task less successfully than young adults as complex motor functions were found to decrease with increasing age (Maes et al., 2017; Ren et al., 2013).

In the context of neurometabolite levels, we found that at rest, neither GABA+, Glx, nor the GABA+/Glx ratio predicted BTT performance. Previous research examining relationships between baseline neurometabolite levels and bimanual performance in SM1 and dorsal premotor cortex (PMd) found no significant associations between baseline metabolites (GABA+ or Glx) and task performance in short-term learning, but a relationship with long-term motor learning over several weeks (Hehl et al., 2025). Additional evidence has been found in other studies investigating age-related GABA+ levels in SM1, in which lower baseline GABA+ levels were associated with better performance (Chalavi et al., 2018; Maes et al., 2022; Stagg et al., 2011). Predictive properties of baseline metabolite concentrations seem inconsistent and dependent on task complexity, ROI, and age group (Li et al., 2022; Maes et al., 2021).

Our findings revealed a significant relationship between task-related GABA+/Glx ratio and BTT performance dependent on age group, with the ratio of GABA+/Glx being negatively associated with better task performance in older adults only. This suggests that older adults seem to benefit from a lower inhibitory tone during bimanual task demands and is in line with previous research investigating GABA+ levels in SM1 (Maes et al., 2022).

In contrast, we found no relationship between task-related Glx levels and bimanual performance using an advanced LCM fitting approach, which accounts for the contribution of glutathione (GSH) to the Glx peak in simple peak fitting approaches. If not corrected for, a direct influence of GSH on motor performance might be possible, particularly in older adults (Hupfeld et al., 2021), potentially affecting the Glx–motor performance relationship. Moreover, since Glx quantification represents a combined signal from glutamate (Glu) and glutamine (Gln), the current results should be viewed within the broader framework of brain energetics. The tightly regulated Glu–Gln cycle complicates the attribution of observed effects specifically to glutamatergic neurotransmission. Gln serves as a precursor to Glu and is closely tied to metabolic processes, including those within the tricarboxylic acid (TCA) cycle. Consequently, Glx measurements may partially reflect these underlying metabolic dynamics (Ramadan et al., 2013).

### 4.4 Limitations and future directions

Due to the relatively large voxel, which was necessary for adequate signal quality of small concentration neurometabolites, we could not differentiate between left and right SMA in the current study. In addition, GABA+ has been quantified in this study, which includes co-edited macromolecules, resulting in potential macromolecular contamination (Harris, Puts, Barker, et al., 2015; Mikkelsen, Harris, et al., 2018). This requires careful interpretation, as the effects observed in our study do not reflect pure GABA, but around 50% of the GABA+ signal may originate from co-edited macromolecules, possibly contributing to signal heterogeneity (Harris et al., 2017; Near et al., 2011). Notably, increased macromolecule levels have been observed in the aging brain, underscoring the need for cautious interpretation of GABA+ signals in older compared with younger adults (Aufhaus et al., 2013; Marjańska et al., 2018). Similarly, because Glx includes both Glu and Gln, its quantification may yield different age-related effects compared with analyzing Glu and Gln separately (Roalf et al., 2020). This distinction may call for further research. We believe that our study provides valuable insights into age-related neurometabolite modulations in SMA since prior research concerning this region is still scarce. Future studies might aim to further entangle the balance between GABAergic and glutamatergic modulations in healthy aging and the role of different brain regions in bimanual coordination under varying levels of task difficulty. Differences in task conditions could not be analyzed in our current analysis due to the averaging of metabolite concentrations across the full block containing the execution of various subtasks. This ensured high data quality and signal-to-noise ratio. Considering task complexity in relation to metabolite modulations and cortical activation patterns of different brain areas constituting the motor network might be an insightful future step to enhance our understanding of the role of neurometabolites in motor control.

## 5 Conclusion

This study demonstrates that aging is associated with lower GABA+ and Glx concentrations in the SMA, yet without altering the relative inhibitory–excitatory balance. Task-related modulations in these neurometabolites were not observed at the group level. However, a lower inhibitory tone was linked to better bimanual coordination in older adults. These findings suggest that, while neurometabolite concentrations decline with age, preserved balance and reduced inhibition in the SMA may play a key role in supporting motor performance in aging, highlighting the need for further investigation into inhibitory–excitatory mechanisms.

## Supplementary Material

Supplementary Material

## Data Availability

The data of this study are not publicly available due to ethical restrictions. It is available on request from the corresponding author.
