# Supplementary material for: Age-related GABA- and glutamatergic differences in SMA during bimanual coordination
Source: Imaging Neurosci (Camb). 2025 Nov 26;3:IMAG.a.1036. doi: 10.1162/IMAG.a.1036 (PMC12658773; doi:10.1162/IMAG.a.1036)
Supplement: Supplementary Material [file IMAG.a.1036_supp.pdf]

**Supplementary Materials**: Age-related GABA- and glutamatergic differences in SMA during bimanual coordination

**Supplement 1: Score calculation of the Bimanual Tracking Task (BTT)**

The BTT task was scored as a percentage from 0-100% based on the participant's performance in each trial. The score was based on movement accuracy, direction, rotation frequency and speed. The preliminary percentage of the score was calculated based on the total number of target points covered by the participant's movement trajectory along the target line, divided by the total number of target points (see Fig. 1D in (Adab et al., 2020)). To penalize parallel movements to the target line, an additional formula was calculated as previously described in Hehl et al. (2025). Here, the preliminary percentage P was additionally multiplied by the distance factor D, which lead to the final score S (Hehl et al., 2025)

$$S = P \cdot D$$

The distance factor D was calculated based on the average distance $\bar{d}$ to the target line across each data point within one trial which was divided by 8 and subtracted from 1.:

$$D = \left(1 - \frac{\bar{d}}{8}\right)$$

In contrast to Hehl et al. (2025), we adjusted the denominator to 8 instead of 5 based on the population's sensitivity of progress in a similar independent dataset. Our sample included both older and younger adults, whereas Hehl et al. (2025) examined only young adults. This adjustment aimed to minimize floor and ceiling effects.

**Supplement 2: MRSinMRS checklist – minimum reporting standards (Lin et al., 2021)**

| | | |
|---|---|---|
| **1.** | **Hardware** | |
| a. | Field strength | 3 T |
| b. | Manufacturer | Philips |
| c. | Model | Achieva dstream |
| d. | RF coils: nuclei (transmit/receive), number of channels, type, body part | 32-channel receiver head coil (Philips, Best, The Netherlands) |
| e. | Additional hardware | N/A |
| **2.** | **Acquisition** | |
| a. | Pulse sequence | HERMES<br>1.9 ppm (GABA), 4.56 ppm (GSH) |
| b. | Volume of interest (VOI) locations | Bilateral SMA |
| c. | Nominal VOI size AP x LR x HF($cm^3$, $mm^3$) | 30 x 30 x 30 $mm^3$ |
| d. | Repetition time ($T_R$), echo time ($T_E$) (ms,s) | $T_R$ = 2000 ms ; $T_E$ = 80 ms |
| e. | Total number of excitations of acquisitions per spectrum | 320 averages |
| f. | Additional sequence parameters (spectral width in Hz, number of spectral points, frequency offsets) | 2000 Hz; 1024 points; N/A |
| g. | Water suppression method | Multiply Optimized Insensitive Suppression Train (MOIST; bandwidth 140 Hz) |
| h. | Shimming method, reference peak, and thresholds for "acceptance of shim" chosen | First-order "pencil-beam" (PB) shimming procedure (Philips) |
| i. | Triggering or motion correction method | N/A |
| **3.** | **Data analysis methods and outputs** | |
| a. | Analysis software | Gannet v3.3.2 / Osprey v.2.9.2 |
| b. | Processing steps deviating from the quoted reference or product | N/A |
| c. | Output measure | Alpha-corrected group normalized in institutional units (i.u.) |
| d. | Quantification references and assumptions, fitting model assumptions | 3-Gaussian using a nonlinear baseline |
| **4.** | **Data quality** | |
| a. | Reported variables (SNR, FWHM) | See Table 1 and 2 of the manuscript |
| b. | Add data exclusion criteria | Visual inspection: out-of-voxel echoes, lipid contamination;<br>If no clear GABA+ or Glx signal could be detected or if Gannet modeling of the data failed;<br>SNR below three standard deviations (SD) from the mean, FWHM exceeded three SD above the mean, Fit Error > 12% |
| c. | Quality measures of postprocessing model fitting (e.g., CRLB, goodness of fit, SD of residual) | Fit Error_GABA_rest: YA, 5.17±1.12 / OA, 5.12±1.21; Fit Error_Glx_rest: YA, 2.62±0.63 / OA, 3.08±0.75; Fit Error_GABA_task: YA, 5.40±1.27 / OA, 6.44±3.40; Fit Error_Glx_task: YA, 2.62±0.54 / OA, 3.41±1.56 |
| d. | Sample spectrum | See Figure 3 of the manuscript |

**Supplement 3: Linear Mixed Model Output**

**S3.1 GABA+ Models**

Formula: GABA ~ group * condition + Water_FWHM + (1 | Subject)

**Model: full_model**

| term | estimate | std.error | t value | df | p.value |
|---|---|---|---|---|---|
| (Intercept) | 2.645 | 0.332 | 7.958 | 57.070 | 0.000 |
| groupYoung | 0.224 | 0.129 | 1.743 | 84.369 | 0.085 |
| conditiontask | -0.138 | 0.103 | -1.345 | 49.218 | 0.185 |
| Water_FWHM | 0.014 | 0.037 | 0.368 | 53.379 | 0.714 |
| groupYoung:conditiontask | 0.093 | 0.141 | 0.661 | 48.501 | 0.512 |

Significance levels: *p < .05, **p < .01, ***p < .001

ANOVA Table:

| term | sumsq | meansq | NumDF | DenDF | F value | p.value |
|---|---|---|---|---|---|---|
| group | 0.795 | 0.795 | 1 | 48.195 | 6.414 | 0.015 |
| condition | 0.211 | 0.211 | 1 | 48.430 | 1.703 | 0.198 |
| Water_FWHM | 0.017 | 0.017 | 1 | 53.379 | 0.136 | 0.714 |
| group:condition | 0.054 | 0.054 | 1 | 48.501 | 0.437 | 0.512 |

Significance levels: *p < .05, **p < .01, ***p < .001

Formula: GABA ~ group + Water_FWHM + (1 | Subject)

**Model: final_model**

| term | estimate | std.error | t value | df | p.value |
|---|---|---|---|---|---|
| (Intercept) | 2.559 | 0.327 | 7.816 | 53.719 | 0.000 |
| groupYoung | 0.274 | 0.107 | 2.552 | 47.988 | 0.014 |
| Water_FWHM | 0.015 | 0.037 | 0.407 | 53.353 | 0.686 |

Significance levels: *p < .05, **p < .01, ***p < .001

ANOVA Table:

| term | sumsq | meansq | NumDF | DenDF | F value | p.value |
|---|---|---|---|---|---|---|
| group | 0.80 | 0.80 | 1 | 47.988 | 6.515 | 0.014 |
| Water_FWHM | 0.02 | 0.02 | 1 | 53.353 | 0.166 | 0.686 |

Significance levels: *p < .05, **p < .01, ***p < .001

## S3.2 Glx Models

Formula: Glx ~ group * condition + water_FWHM + (1 | subject)

**Model: full_model**

| term | estimate | std.error | statistic | df | p.value |
|---|---|---|---|---|---|
| (Intercept) | 19.95 | 1.51 | 13.19 | 75.41 | 0.00 |
| groupYA | 4.60 | 0.83 | 5.52 | 69.71 | 0.00 |
| conditiontask | 0.84 | 0.52 | 1.64 | 48.07 | 0.11 |
| water_FWHM | -0.38 | 0.27 | -1.42 | 76.49 | 0.16 |
| groupYA:conditiontask | -0.55 | 0.70 | -0.79 | 47.61 | 0.43 |

Significance levels: *p < .05, **p < .01, ***p < .001

ANOVA Table:

| term | sumsq | meansq | NumDF | DenDF | statistic | p.value |
|---|---|---|---|---|---|---|
| group | 97.11 | 97.11 | 1.00 | 50.90 | 31.73 | 0.00 |
| condition | 7.75 | 7.75 | 1.00 | 49.73 | 2.53 | 0.12 |
| water_FWHM | 6.20 | 6.20 | 1.00 | 76.49 | 2.03 | 0.16 |
| group:condition | 1.90 | 1.90 | 1.00 | 47.61 | 0.62 | 0.43 |

Significance levels: *p < .05, **p < .01, ***p < .001

Formula: Glx ~ group + water_FWHM + (1 | subject)

**Model: final_model**

| term | estimate | std.error | statistic | df | p.value |
|---|---|---|---|---|---|
| (Intercept) | 20.01 | 1.49 | 13.45 | 75.96 | 0.00 |
| groupYA | 4.26 | 0.76 | 5.57 | 51.53 | 0.00 |
| water_FWHM | -0.32 | 0.26 | -1.20 | 80.16 | 0.23 |

Significance levels: *p < .05, **p < .01, ***p < .001

ANOVA Table:

| term | sumsq | meansq | NumDF | DenDF | statistic | p.value |
|---|---|---|---|---|---|---|
| group | 97.12 | 97.12 | 1.00 | 51.53 | 31.07 | 0.00 |
| water_FWHM | 4.49 | 4.49 | 1.00 | 80.16 | 1.44 | 0.23 |

Significance levels: *p < .05, **p < .01, ***p < .001

## Supplement 4: Multiple Linear Regression Output

### S4.1 Resting State Models

Formula: Score8 ~ group * (GABA_rest_gan + Glx_rest)

**Model: model_rest**

| term | estimate | std.error | statistic | p.value |
|---|---|---|---|---|
| (Intercept) | 47.774 | 24.262 | 1.969 | 0.056 |
| groupYA | -26.233 | 37.456 | -0.700 | 0.488 |
| GABA_rest_gan | -2.439 | 6.927 | -0.352 | 0.727 |
| Glx_rest | -0.352 | 0.899 | -0.392 | 0.697 |
| groupYA:GABA_rest_gan | -1.253 | 9.424 | -0.133 | 0.895 |
| groupYA:Glx_rest | 2.276 | 1.436 | 1.585 | 0.121 |

ANOVA Type 3 Table:

| Sum Sq | Df | F value | Pr(>F) |
|---|---|---|---|
| 597.806 | 1 | 3.877 | 0.056 |
| 75.630 | 1 | 0.491 | 0.488 |
| 19.110 | 1 | 0.124 | 0.727 |
| 23.669 | 1 | 0.154 | 0.697 |
| 2.724 | 1 | 0.018 | 0.895 |
| 387.580 | 1 | 2.514 | 0.121 |
| 6,321.559 | 41 | | |

Formula: Score8 ~ group

**Model: model_rest4**

| term | estimate | std.error | statistic | p.value |
|---|---|---|---|---|
| (Intercept) | 34.560 | 2.515 | 13.740 | 0 |
| groupYA | 18.777 | 3.453 | 5.438 | 0 |

ANOVA Type 3 Table:

| Sum Sq | Df | F value | Pr(>F) |
|---|---|---|---|
| 27,471.073 | 1 | 188.794 | 0 |
| 4,303.065 | 1 | 29.573 | 0 |
| 6,838.884 | 47 | | |

## S4.2 Task-related models

Formula: Score8 ~ group * (GABA_task_gan + Glx_task) + water_FWHM_gan_task

**Model: model_task**

| term | estimate | std.error | statistic | p.value |
|---|---|---|---|---|
| (Intercept) | 54.496 | 23.175 | 2.351 | 0.023 |
| groupYA | -30.606 | 29.911 | -1.023 | 0.312 |
| GABA_task_gan | -9.359 | 5.192 | -1.802 | 0.079 |
| Glx_task | 0.195 | 0.871 | 0.223 | 0.824 |
| water_FWHM_gan_task | 0.124 | 1.661 | 0.075 | 0.941 |
| groupYA:GABA_task_gan | 5.362 | 7.384 | 0.726 | 0.472 |
| groupYA:Glx_task | 1.597 | 1.226 | 1.303 | 0.200 |

ANOVA Type 3 Table:

| Sum Sq | Df | F value | Pr(>F) |
|---|---|---|---|
| 760.441 | 1 | 5.530 | 0.023 |
| 143.995 | 1 | 1.047 | 0.312 |
| 446.815 | 1 | 3.249 | 0.079 |
| 6.865 | 1 | 0.050 | 0.824 |
| 0.770 | 1 | 0.006 | 0.941 |
| 72.521 | 1 | 0.527 | 0.472 |
| 233.558 | 1 | 1.698 | 0.200 |
| 5,776.020 | 42 | | |

Formula: Score8 ~ group * (GABA_task_gan) + Glx_task + water_FWHM_gan_task

**Model: model_task1**

| term | estimate | std.error | statistic | p.value |
|---|---|---|---|---|
| (Intercept) | 43.032 | 21.614 | 1.991 | 0.053 |
| groupYA | -2.586 | 20.961 | -0.123 | 0.902 |
| GABA_task_gan | -9.856 | 5.220 | -1.888 | 0.066 |
| Glx_task | 1.009 | 0.612 | 1.650 | 0.106 |
| water_FWHM_gan_task | -0.190 | 1.657 | -0.115 | 0.909 |
| groupYA:GABA_task_gan | 7.147 | 7.315 | 0.977 | 0.334 |

ANOVA Type 3 Table:

| Sum Sq | Df | F value | Pr(>F) |
|---|---|---|---|
| 553.968 | 1 | 3.964 | 0.053 |
| 2.128 | 1 | 0.015 | 0.902 |
| 498.191 | 1 | 3.565 | 0.066 |
| 380.321 | 1 | 2.721 | 0.106 |
| 1.838 | 1 | 0.013 | 0.909 |
| 133.398 | 1 | 0.954 | 0.334 |
| 6,009.578 | 43 | | |

Formula: Score8 ~ group + water_FWHM_gan_task

**Model: model_task4**

| term | estimate | std.error | statistic | p.value |
|---|---|---|---|---|
| (Intercept) | 38.956 | 13.959 | 2.791 | 0.008 |
| groupYA | 19.221 | 3.751 | 5.124 | 0.000 |
| water_FWHM_gan_task | -0.539 | 1.682 | -0.320 | 0.750 |

ANOVA Type 3 Table:

| Sum Sq | Df | F value | Pr(>F) |
|---|---|---|---|
| 1,155.326 | 1 | 7.788 | 0.008 |
| 3,894.407 | 1 | 26.253 | 0.000 |
| 15.214 | 1 | 0.103 | 0.750 |
| 6,823.670 | 46 | | |

Formula: Score8 ~ group * (GABAGlx_rest_Osp)

## S4.3 GABA+/Glx ratio models: resting state

Formula: Score8 ~ group * (GABAGlx_rest_Osp)

Model: model_ratio_rest

| term | estimate | std.error | statistic | p.value |
|---|---|---|---|---|
| (Intercept) | 42.746 | 5.736 | 7.452 | 0.000 |
| groupYA | 10.503 | 8.255 | 1.272 | 0.210 |
| GABAGlx_rest_Osp | -9.967 | 6.285 | -1.586 | 0.120 |
| groupYA:GABAGlx_rest_Osp | 10.098 | 10.242 | 0.986 | 0.329 |

ANOVA Type 3 Table:

| Sum Sq | Df | F value | Pr(>F) |
|---|---|---|---|
| 7,992.846 | 1 | 55.533 | 0.000 |
| 233.006 | 1 | 1.619 | 0.210 |
| 362.000 | 1 | 2.515 | 0.120 |
| 139.911 | 1 | 0.972 | 0.329 |
| 6,476.847 | 45 | | |

Formula: Score8 ~ group

**Model: model_ratio_rest2**

| term | estimate | std.error | statistic | p.value |
|---|---|---|---|---|
| (Intercept) | 34.560 | 2.515 | 13.740 | 0.000 |
| groupYA | 18.777 | 3.453 | 5.438 | 0.000 |

ANOVA Type 3 Table:

| Sum Sq | Df | F value | Pr(>F) |
|---|---|---|---|
| 27,471.073 | 1 | 188.794 | 0.000 |
| 4,303.065 | 1 | 29.573 | 0.000 |
| 6,838.884 | 47 | | |

## S4.4 GABA+/Glx ratio models: task-related

Formula: Score8 ~ group * (GABAGlx_task_Osp) + water_FWHM_gan_task

**Model: model_ratio_task**

| term | estimate | std.error | statistic | p.value |
|---|---|---|---|---|
| (Intercept) | 53.489 | 14.260 | 3.751 | 0.001 |
| groupYA | 2.512 | 8.410 | 0.299 | 0.767 |
| GABAGlx_task_Osp | -26.182 | 9.522 | -2.750 | 0.009 |
| water_FWHM_gan_task | -0.370 | 1.594 | -0.232 | 0.818 |
| groupYA:GABAGlx_task_Osp | 27.183 | 12.007 | 2.264 | 0.029 |

ANOVA Type 3 Table:

| Sum Sq | Df | F value | Pr(>F) |
|---|---|---|---|
| 1,861.526 | 1 | 14.070 | 0.001 |
| 11.808 | 1 | 0.089 | 0.767 |

| Sum Sq | Df | F value | Pr(>F) |
|---|---|---|---|
| 1,000.190 | 1 | 7.560 | 0.009 |
| 7.117 | 1 | 0.054 | 0.818 |
| 678.087 | 1 | 5.125 | 0.029 |
| 5,821.254 | 44 | | |

**Supplement 5**

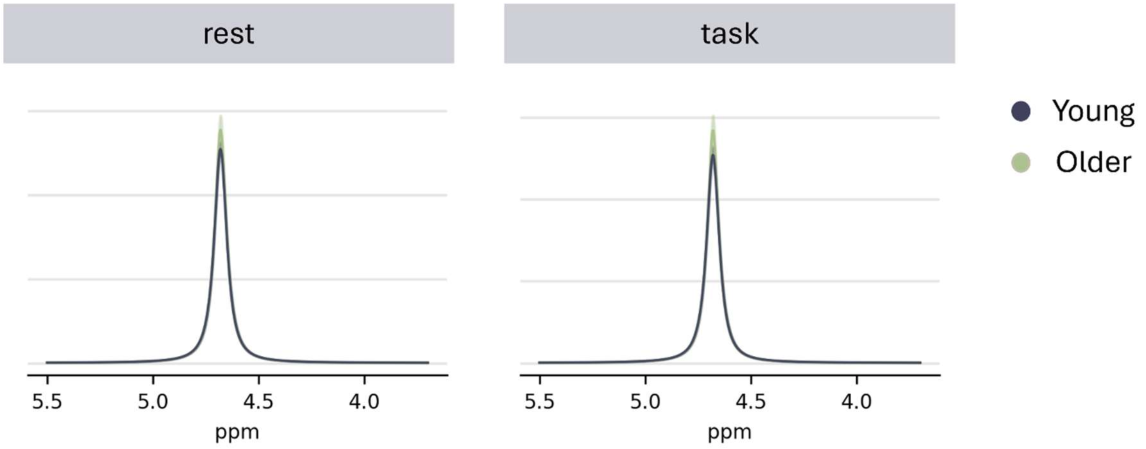


**Figure S1**: MRS spectra (mean; 95% CI) of water peaks per group and condition.

## References

Adab, H. Z., Chalavi, S., Monteiro, T. S., Gooijers, J., Dhollander, T., Mantini, D., & Swinnen, S. P. (2020). Fiber-specific variations in anterior transcallosal white matter structure contribute to age-related differences in motor performance. *Neuroimage*, *209*, 116530.

Hehl, M., Malderen, S. V., Blashchuk, S., Sunaert, S., Edden, R. A., Swinnen, S. P., & Cuypers, K. (2025). The Reciprocal Relationship Between Short-and Long-Term Motor Learning and Neurometabolites. *Human brain mapping*, *46*(4), e70170.
